# Deep Neck Space Involvement in *Burkholderia pseudomallei* Infection

**DOI:** 10.31662/jmaj.2026-0120

**Published:** 2026-08-07

**Authors:** Chee Yik Chang, Yong Wang Su

**Affiliations:** 1 Infectious Disease Unit, Department of Medicine, Hospital Sultanah Aminah, Johor, Malaysia; 2 Department of Medicine, Hospital Sultanah Aminah, Johor, Malaysia

**Keywords:** melioidosis, *Burkholderia pseudomallei*, deep neck space infection

## To the Editor,

We read with great interest the article by Mohanty et al. describing melioidosis presenting as a parapharyngeal abscess ^(1)^. The authors highlighted an uncommon manifestation of *Burkholderia pseudomallei* infection involving the deep neck space. This shares certain similarities with our previously reported case and emphasizes the diagnostic challenges associated with atypical presentations of melioidosis ^(2)^.

Melioidosis is endemic in many tropical regions, including Southeast Asia and northern Australia, and is well known for its broad spectrum of clinical manifestations. The most common presentations include pneumonia, bacteremia, and visceral abscesses. In contrast, involvement of the head and neck region is relatively uncommon. Nevertheless, accumulating evidence indicates that *B. pseudomallei* can cause a variety of cervicofacial infections. A retrospective study of head and neck melioidosis demonstrated that parotid abscess, neck abscess, and suppurative lymphadenitis were the predominant clinical manifestations ^(3)^. Case reports from India and Southeast Asia also describe rare presentations of melioidosis involving deep neck spaces, including retropharyngeal and parapharyngeal abscesses. These infections may mimic other conditions, such as tuberculous lymphadenitis or pyogenic abscesses, often leading to delays in diagnosis ^(4)^.

Host factors, such as diabetes mellitus, chronic kidney disease, or immunosuppression, can impair neutrophil function and cell-mediated immunity, allowing the bacterium to survive and replicate in tissues. Involvement of deep neck spaces, such as the parapharyngeal, retropharyngeal, and submandibular regions, can result from hematogenous seeding, lymphatic spread from oropharyngeal or odontogenic foci, or direct contiguous extension. Within these potential spaces, *B. pseudomallei* induces granulomatous inflammation and tissue necrosis, leading to pus accumulation and abscess formation ^(5)^.

The diagnosis of melioidosis relies on microbiological confirmation, with culture remaining the gold standard. Isolation of *Burkholderia pseudomallei* from clinical specimens such as blood, pus, sputum, or other body fluids establishes the diagnosis. Rapid identification methods, including matrix-assisted laser desorption ionization–time of flight mass spectrometry, have improved the speed and accuracy of organism identification. Polymerase chain reaction-based assays provide rapid and highly sensitive detection of *B. pseudomallei*, although access to these assays remains limited in many endemic settings ^(6)^.

Melioidosis may involve various deep neck spaces, including the parapharyngeal and retropharyngeal regions. Clinicians practicing in endemic areas should therefore consider melioidosis in the differential diagnosis of unusual neck abscesses, particularly in patients with risk factors. Early recognition through microbiological culture and timely initiation of appropriate antimicrobial therapy remain essential for optimal clinical outcomes.

## Article Information

### Author Contributions

Conception and design of the study, acquisition of data, drafting the article, final approval of the version to be submitted, supervision: Chee Yik Chang. Acquisition of data, drafting the article, final approval of the version to be submitted: Yong Wang Su.

### Conflicts of Interest

None

### Consent Statement

Not required.
